# Minimal Change Disease Associated with High-dose Aspirin

**DOI:** 10.7759/cureus.3408

**Published:** 2018-10-04

**Authors:** Vaibhav Rastogi, Shreyans Doshi, Ayesha Kaleem

**Affiliations:** 1 Internal Medicine, University of Central Florida, Orlando, USA; 2 Nephrology, North Florida Regional Medical Center, Gainesville, USA

**Keywords:** minimal change disease, acute tubular necrosis, aspirin, complication

## Abstract

Minimal change disease (MCD) is an etiology of nephrotic syndrome that is more common in the pediatric population as compared to the adult population. Steroids are an effective treatment for MCD. Non-steroidal anti-inflammatory drugs (NSAIDS) are well known for their nephrotoxicity when used chronically. However, there are only few cases of NSAIDS-induced MCD that have been reported in the literature. Our patient is a 72-year-old male with no significant past medical history who presented with shortness of breath, fatigue, and malaise for few weeks. His renal function was declining in the hospital despite renal protective therapies. His medication history was significant for chronic BC powder (high dose aspirin with caffeine) use. Renal biopsy was performed and showed MCD and acute tubular necrosis. Steroids were initiated and patient’s kidney function improved.

## Introduction

Minimal change disease (MCD) or minimal change glomerulopathy is the most prevalent etiology of idiopathic nephrotic syndrome in children and accounts for 10%-15% of cases in adults. The common presentation includes edema and weight gain due to fluid retention that is acute in nature. Labs usually comprise elevated urinary protein and azotemia. It is generally not associated with urinary casts. Renal biopsy is the gold standard for MCD. Light and immunofluorescence microscopy shows normal kidney or may reveal only mild mesangial cell proliferation [1]. It is the electron microscopy which affirms the diagnosis as it demonstrates diffuse effacement of the epithelial cell foot processes. It is steroid-sensitive nephrotic syndrome with a typical response time of 8-16 weeks. However, relapse can be seen frequently. In approximately 40% of patients, the course of MCD is one of remission followed by relapse [1]. Here, we describe an adult case of MCD attributable to long-term use of non-steroidal anti-inflammatory drugs (NSAIDs).

## Case presentation

A 72-year-old Caucasian male with no significant past medical history presented with fatigue, shortness of breath, and malaise for a couple of weeks. He had paroxysmal nocturnal dyspnea, leg swelling and gained 26 pounds in two weeks. Physical exam was positive for crackles at bilateral lung bases and bilateral pedal edema. His home medications included only BC powder (high-dose aspirin with caffeine) on a daily basis for headaches for few months.

**Figure 1:**
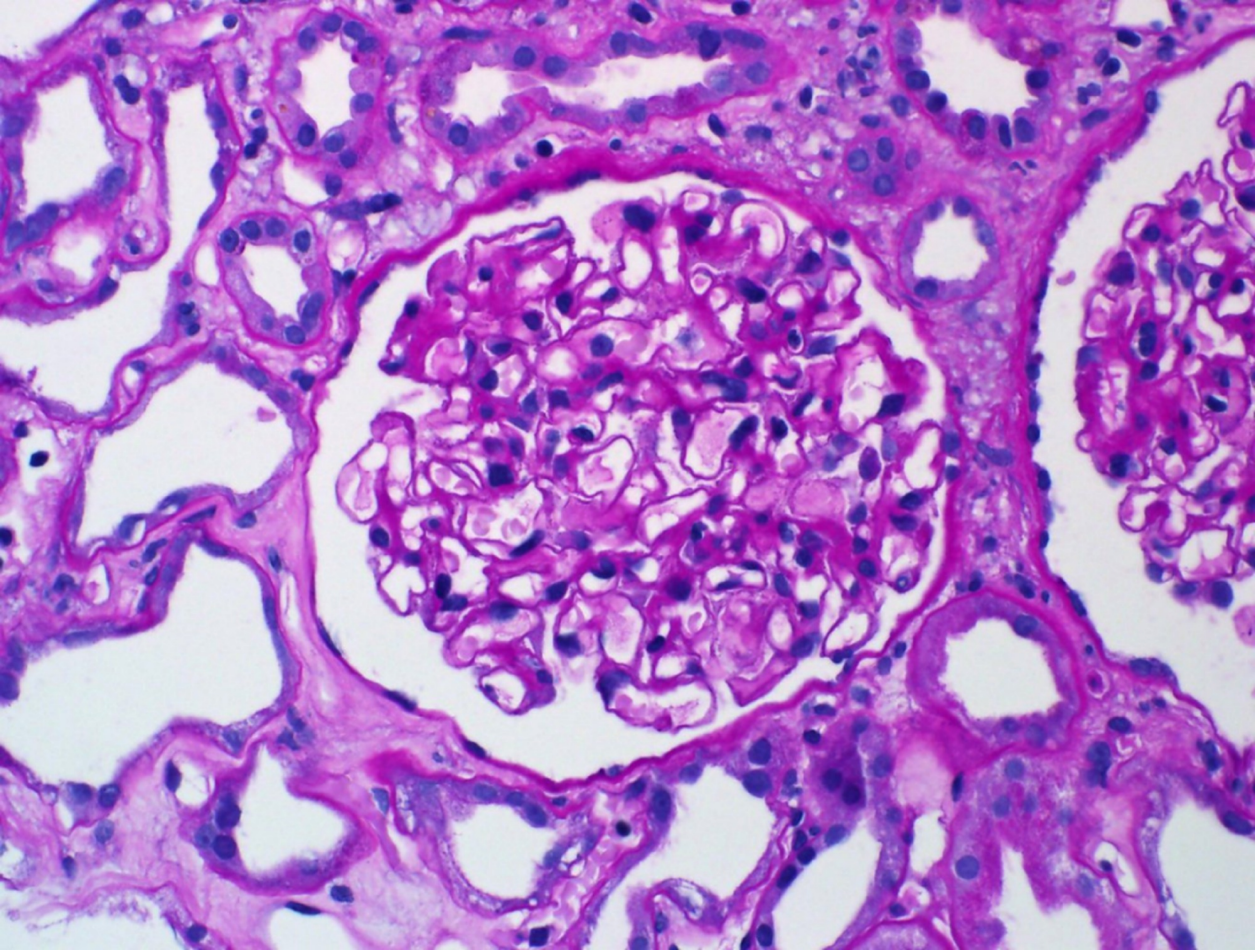
Light microscopy showed that glomeruli have open capillary loops with no evidence of cellular crescents, fibrinoid necrosis, or endocapillary hypercellularity. The tubulointerstitial compartment is marked by severe acute tubular injury, with tubular dilatation, epithelial simplification, cytoplasmic vacuolization, and nuclear reactive changes. There is mild interstitial edema and patchy inflammatory infiltrate.

**Figure 2:**
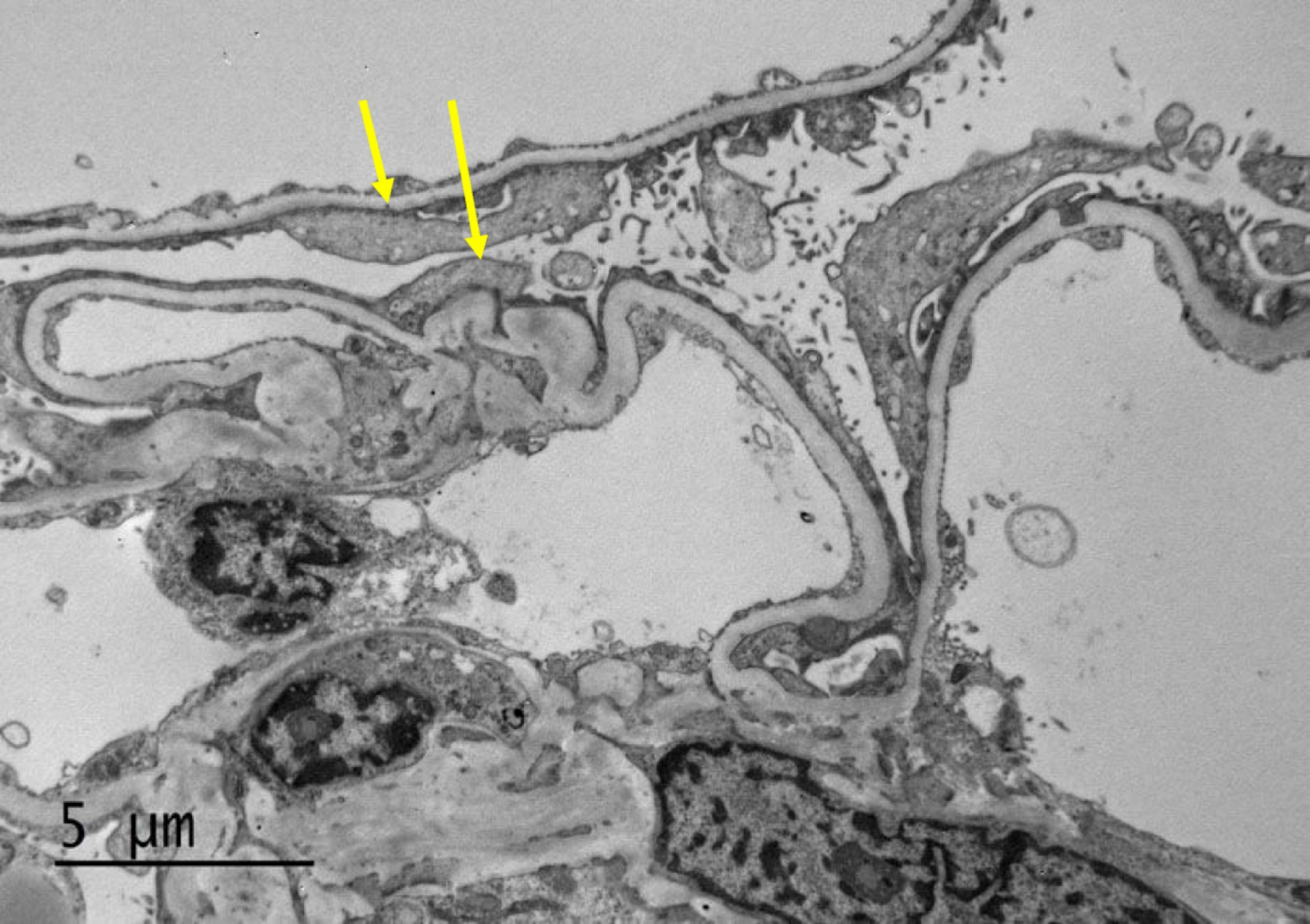
Electron microscopy showed diffuse effacement of podocyte foot processes (arrows). The capillary loop basement membranes are uniform and of normal thickness. There is no capillary loop hypercellularity or sclerosis and no electron-dense deposits are identified. The mesangial matrix is not expanded and no hypercellularity or electron-dense deposits are present. The tubular basement membranes do not show evidence of immune-type deposits.

His blood urea nitrogen (BUN) and creatinine levels on admission were 25 and 1.62 mg/dl. Lower extremity ultrasound showed a right popliteal vein deep venous thrombosis. Ventilation/perfusion scan of lungs was of intermediate probability. He was started on an anticoagulation regimen. His low density lipoprotein (LDL) was 233 mg/dl. Progressive elevation was noticed in his BUN and creatinine during the hospital stay despite institution of protective therapies including fluids. Urinalysis showed proteinuria with no hematuria or pyuria. His 24-hour urine protein was 16.3 g and urine protein/creatinine ratio was 10.2. Renal ultrasound was unremarkable. Urine sodium was 14. Serum protein electrophoresis showed low serum protein and albumin with high alpha-2 globulin and low gamma globulin. Urine protein electrophoresis showed a total protein concentration of 1414.6 mg/dl. Anti-phospholipase A2 receptor antibody was negative. Anti-nuclear antibody, hepatitis B surface antigen and antibody, hepatitis C antibody, and human immunodeficiency virus (HIV) test were negative. Complement C3 and C4 levels were normal. Beta-2-microglobulin was high. Kappa and lambda chains were both high and the ratio was 1.73. Abdominal fat pad biopsy was negative for amyloidosis. The patient was later started on hemodialysis due to worsening renal function. Renal biopsy was done. Light microscopy showed severe acute tubular injury with tubular dilatation, epithelial simplification, cytoplasmic vacuolization, nuclear reactive changes, mild interstitial edema, and mild interstitial fibrosis (Figure 1). Electron microscopy showed diffuse effacement of podocyte foot processes (Figure 2). Thus, the diagnosis of MCD with severe acute tubular necrosis was made based on the biopsy results. The patient was started on prednisone. At discharge, patient was instructed to stop using BC powder. The kidney function improved within six weeks of treatment institution and dialysis was stopped.

## Discussion

Nephrotoxicity of non-steroidal anti-inflammatory drugs (NSAIDs) has been widely described. They can cause acute tubular necrosis, acute tubulointerstitial nephritis, glomerulonephritis, and chronic renal failure. Others include renal papillary necrosis, hypertension, and hyperreninemic hypoaldosteronism. NSAIDs nonselectively inhibit cyclooxygenases in the arachidonic acid pathway. This disrupts the production of prostaglandins which results in vasoconstriction [2]. It also causes shunting of arachidonic acid to lipoxygenase pathway which in turn ensures elevated levels of leukotrienes in the body. Leukotrienes are pro-inflammatory as well as vasoconstrictive. In normal healthy adults, vasoconstriction in renal capillary bed with predilection towards afferent arterioles culminates with decrease in glomerular filtration rate. However, during conditions that cause hypoperfusion of kidney such as congestive heart failure, hypovolemia and end stage liver cirrhosis, it can result in acute renal failure [3].

This is the first case of MCD and acute tubular necrosis due to high-dose aspirin (analgesic dose) that has been documented in the literature in adults. There are only a few cases of NSAID-induced MCD in adults that have been described in the literature [4-5]. The exact pathogenesis of NSAIDs in MCD is not clear. T-cells have been implicated in the podocyte injury especially CD8 T suppressor cells being predominantly present during active disease. Increased levels of cytokines associated with T helper cells including interleukin (IL)-2, IL-4, IL-5, IL-9, IL-10, and IL-13 have also been seen in MCD [1]. Prostaglandin E2 has immunosuppressive effects and impedes the activation and proliferation of these T-cells. NSAIDs inhibit prostaglandin E2 synthesis. This potentiates the activation of T-cells and release of T-cell-mediated cytokines which may ultimately result in podocyte injury. Another mechanism might be the leukotrienes-mediated enhancement of vascular permeability. This modifies the glomerular-capillary barrier which will facilitate proteinuria [3].

Immune suppression is the treatment of choice as immune mediators are involved in the pathogenesis of MCD. Steroids are the first mode of treatment, followed by steroid-sparing agents including alkylating agents or calcineurin inhibitors [1].

## Conclusions

Long-term analgesic dose aspirin intake can result in MCD. Physicians should have it in their differentials in nephrotic syndrome patients who are chronically on high-dose aspirin.
